# The prevalence of cardiovascular disease in Ethiopia: a systematic review and meta-analysis of institutional and community-based studies

**DOI:** 10.1186/s12872-020-01828-z

**Published:** 2021-01-18

**Authors:** Dessie Abebaw Angaw, Rahma Ali, Afework Tadele, Shegaye Shumet

**Affiliations:** 1 Department of Epidemiology and Biostatistics, Institute of Public Health, College of Medicine and Health Science, University of Gondar, Gondar, Ethiopia; 2 Department of Population and Family Health, Faculty of Public, Jimma University, Jimma, Ethiopia; 3 Department of Psychiatry, College of Medicine and Health Science, University of Gondar, Gondar, Ethiopia

**Keywords:** Cardiovascular disease, Epidemiology, Ethiopia, Systematic review and meta-analysis

## Abstract

**Background:**

Worldwide cardiovascular disease is the major cause of disability and premature death. This is due to the ascending trend of consuming an unhealthy diet and obesity which increases the risk of hypertension and type 2 diabetes mellitus. Thus this study aimed to determine the pooled prevalence of the cardiovascular disease in Ethiopia.

**Methods:**

Medline, Scopus, and Google Scholar search engines were accessed using medical subject heading (MeSH) terms for studies based in Ethiopia, from 2000 to 2018. However, studies done among a specific group of the population were excluded from the study. Data were extracted by one reviewer and then checked independently by a second reviewer. Studies were qualitatively synthesis in terms of design, quality, study population, outcomes, and result. Sub-group analysis and sensitivity tests were conducted to identify potential influences on the prevalence estimates. Quantitative results were pooled in a statistical meta-analysis using STATA version 14 software.

**Result:**

Nine eligible cross-sectional studies were included in the analysis. The prevalence ranges from 1 to 20%. The pooled prevalence of cardiovascular disease (CVD) was 5% (95% CI: 3–8%). The prevalence was higher in the population who visits hospitals, 8% (95% CI: 4–12%) compared to the general population, 2% (95% CI: 1–5%). There was no significant difference in the overall prevalence of CVD between males and females.

**Conclusion:**

The prevalence of cardiovascular disease was high. A higher prevalence of CVD was found among patients who visited health institutions than the general population and no observed significant sex difference in the prevalence

## Background

Globally, non-communicable disease-related mortality remains high. Cardiovascular disease (CVD), cancer, chronic respiratory disease, and diabetes mellitus are on raising and the leading threat to human health and development. It causes about 35 million deaths each year, of which 85% are in developing countries [1, 2].

CVDs are a cluster of diseases and injuries that affect the cardiovascular system and supporting structures. The main CVDs include (but are not limited to) coronary heart disease, congestive heart failure, angina, peripheral arterial disease, deep vein thrombosis (DVT), and stroke [3, 7].

CVDs are the major cause of disability and premature death. This substantially contributes to the escalating costs of health care [3–5]. Studies showed that the percentage of premature death from CVDs ranges from 4% in high-income countries to 42% in low-income countries, depicting growing inequalities among populations based in different countries [6].

The burden is now growing faster than our capacity to combat it and the prevalence is high among people with obesity, poor diet, high blood pressure and type 2 diabetes [8–10] The burden is now growing faster than our capacity to combat it and is the prevalence is high among people with obesity, poor diet, high blood pressure and type 2 diabetes [8–10]. Even though CVD is preventable, about 31% of all global deaths are attributed to CVD [4], and over 3 million deaths occurred before the age of 60 years. Over 80% of CVD associated deaths were in low-and middle-income countries. According to the global disease burden report 2015, the growth and aging of the population have increased the proportion of deaths resulted from CVD in many poorer regions of the world. The disease has a high rate in Eastern and Central Sub-Saharan Africa compared to Western and Southern Sub-Saharan Africa [13].

The contributing factors to CVD are multifarious, including smoking tobacco, hypercholesterolemia, diabetes, sedentary lifestyle, overweight/obesity, energy-dense diet, excessive alcohol consumption, age, sex, family history, and ethnicity [6, 7].

The prevalence of CVD is notoriously difficult to estimate in a population because it requires information about those who do not visit the health facility. Estimating the global prevalence of CVD is challenging due to multiple countries that are reporting the prevalence ascertained with a varying methodology which renders interpretation difficult [8]. Likewise, estimating the burden of CVD is a challenging task in sub-Saharan Africa countries including Ethiopia [13]. In 2014, the World Health Organization reported that around 30% of the Ethiopian population died due to non-communicable diseases, of which, CVD contributes 9% [1].

A systematic review conducted in Ethiopia found that the prevalence of CVD ranges from 7.2 to 24% [10]. Though this study provides a general indication about CVD prevalence, it did not calculate the pooled prevalence of other NCDs and the geographic variation in CVD prevalence is not known in Ethiopia. Therefore, our review aimed to show the pattern and pooled prevalence of CVD with a subgroup analysis of CVD based on regions, sex, and population type included in the primary study (hospital and community based). Our findings on the prevalence and the pattern of CVD in Ethiopia may have important implications for healthcare planning and for the provision of health care services.

## Methods

### Objectives

The primary objective of this review was to assess the quantitative pooled of CVD prevalence and the secondary objective was identifying the effect of sex on cardiovascular disease and investigating any regional differences in Ethiopia.

### Eligibility criteria

We settled the following criteria to incorporate studies in the review: (1) community or institution-based studies conducted in Ethiopia; (2) cross-sectional study with clear objectives and methods; (3) articles between the year 2000 and 2018; (4) articles which address the prevalence of at least one form of CVD like stroke, coronary heart disease, rheumatic heart disease, and congenital heart disease. However, studies among specific sex groups were excluded.

### Search strategy

An extensive search of the literature in databases (Medline and SCOPUS) and a search engine, Google Scholar was done. The initial search was done by scholars having with broad experience in systematic reviews, and screening of titles, abstracts, and full-texts were conducted independently by two reviewers (DA & RA). In the case of disagreements, the third reviewer (SH) was invited and involved to reach a consensus.

The initial search terms were cardiovascular disease, stroke (cerebrovascular accident, cerebral stroke, and cerebrovascular apoplexy), hypertensive heart disease (high blood pressure, vascular resistance), heart failure (cardiac failure, congestive heart failure, heart decomposition, right/left heart failure and myocardial failure), and Ethiopia (Additional file 1).

In the searching strategy, a combination of keywords related to cardiovascular disease, terms related to study design (prevalence, epidemiology, cross-sectional study, clinical/hospital-based, community-based, and population), and title, title/abstract, or medical subject heading was developed (Additional file 1). Additional relevant articles were identified byways of searching the reference lists of full-text articles and grey literature from the institution's websites.

### Risk of bias assessment

The selection of the articles was based on the standardized critical appraisal instrument adapted from Hoy et al.'s risk of bias tool [11]. The tool has 9 items, with a maximum score of nine and a minimum of zero. The overall risk of the bias has been leveled into three categories: 0–3 = low risk, 4–6 = moderate risk, and 7–9 = high risk.

### Data extraction and outcome of interest

Two authors (DA & RA) extract the data, and they have compared the results. Discrepancies were resolved by discussion, or the third reviewer made the decision. The primary authors of the eligible studies were contacted through their email or phone for further clarification about the data. We extracted the following data from each study:

(i) Author(s) and years of publication
(ii) Study design (cross-sectional)
(iii) Country of region and participants (children, adults or older)
(iv) Prevalence estimates reported stratified by age, sex, or location.

The primary outcomes were the population/community-based prevalence of CVD and clinical/hospital-based prevalence of CVD. The secondary outcomes were the prevalence of CVD among males and females.

### Reliability

The second reviewer (RA) was blinded to the primary reviewer’s (DA) decisions on article selection, data extraction, and risk of bias assessment. Any differences were solved by discussion; otherwise, a third reviewer (AT) was available to arbitrate any issues that remained unresolved.

### Analysis of the data

An initial descriptive analysis of the studies has been employed. Heterogeneity between estimates was assessed using the I^2^ statistic, An I^2^ value of above 75% indicates considerable heterogeneity [12].

Potential influences on the prevalence estimate were investigated using sensitivity analyses. Where studies allowed, we descriptively compared prevalence estimates by the source of the population (general/hospital), sex, and regions of the country. Quantitative papers were pooled in a statistical meta-analysis using STATA version14.

## Result

### The review processes

**Fig. 1:**
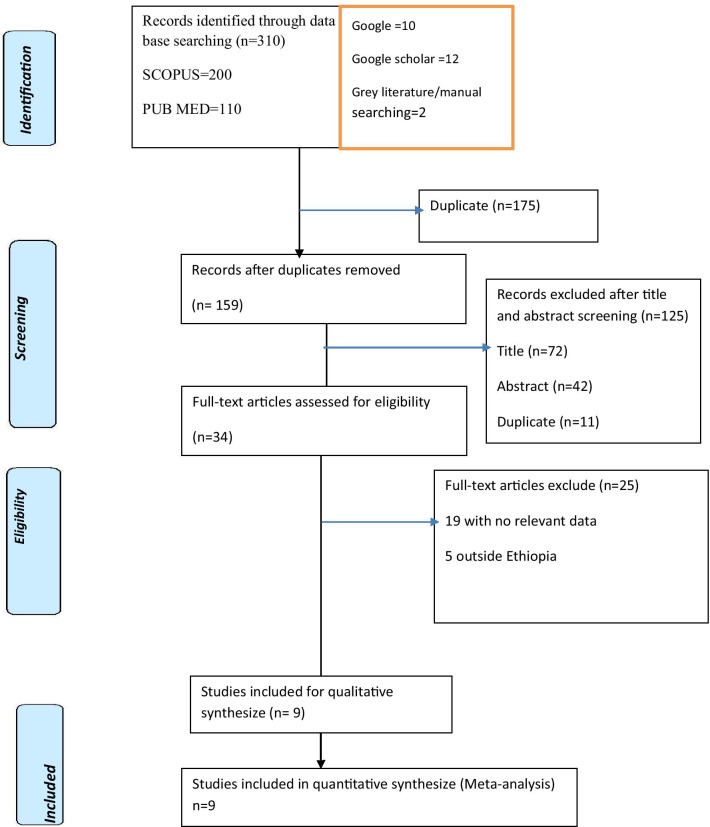
PRISMA flow chart for identifications of studies those were included in systematic review and meta-analysis

The initial database search generated 334 articles. After the removal of duplicates by the title and abstract, 34 remained and considered in the full-text review. Then, the full-text of 25 articles was excluded and nine articles were included for both the systematic review and meta-analysis (Fig. 1).

### Characteristics of the included studies

A total of nine studies with 125,389 participants have been included. About a quarter (33.3%) of the studies based in the Oromia region [13–17], the Southern Nation Nationalities and People (SNNPs) and Amhara each contributed two studies [14, 21], one in Addis Ababa [18], and one study conducted in all regions of the country [19] were included. All the included studies were cross-sectional and published between 2008 [15] and 2018 [20].

From the nine included studies four studies were from community-based [13, 14, 16, 19] and the rest were hospital-based [15, 17, 18, 21]. Three studies reported the rheumatoid heart disease (RHD) form of CVD, one stroke and the rest were general CVD (Table 1). To diagnose CVD, the international classification of disease (ICD-10) and standardized evidence-based echocardiographic (ECG) criteria of the world heart federation was used.

**Table 1:** Characteristics of the individual studies included in this systematic review and meta-analysis

References	Study population	Region _Ethiopia	Diagnosis	Total sample size (N)	Diseased (n)	Proportion (n/N)
Accorsi et al. [15]	Hospital based	Oromia	CVD	22,377	642	2.8%
Deresse et al. [21]	HOSPITAL-based	SNNPS	CVD (stroke)	1471	201	13.7%
Engel et al. [14]	Community-based	Oromia	CVD (RHD)	2000	61	3%
Gebremariam and Moges [18]	Hospital-based	Addis Ababa	CVD	3672	106	2.9%
Gemech et al. [13]	Community-based	Oromia	RHD	987	56	5.7%
Gordon et al. [17]	Hospital-based	Amhara	CVD	1927	392	20.3%
Abebe et al. [16]	community based	Amhara	CVD	67,397	404	0.6%
Yadeta et al. [19]	Community-based	National	CVD (RHD)	3238	59	1.8%
Endriyas et al. [20]	Hospital-based	SNNP	CVD	22,320	1246	5.6

### Risk of bias

A summary of the risk of bias for all the nine included articles with a justification of rating for each item is provided in the supplementary appendix (Additional file 2).

### Assessment of publication bias

Publication bias was assessed using Egger’s test. The estimated bias coefficient was 0.03 (Egger bias B = 0.03 (95% CI: 0.02–0.11; *p* = 0.38)) with a standard error of 0.03. The test thus provides no evidence for the presence of small-study effects (Additional file 3).

### The prevalence of the cardiovascular disease

**Fig. 2:**
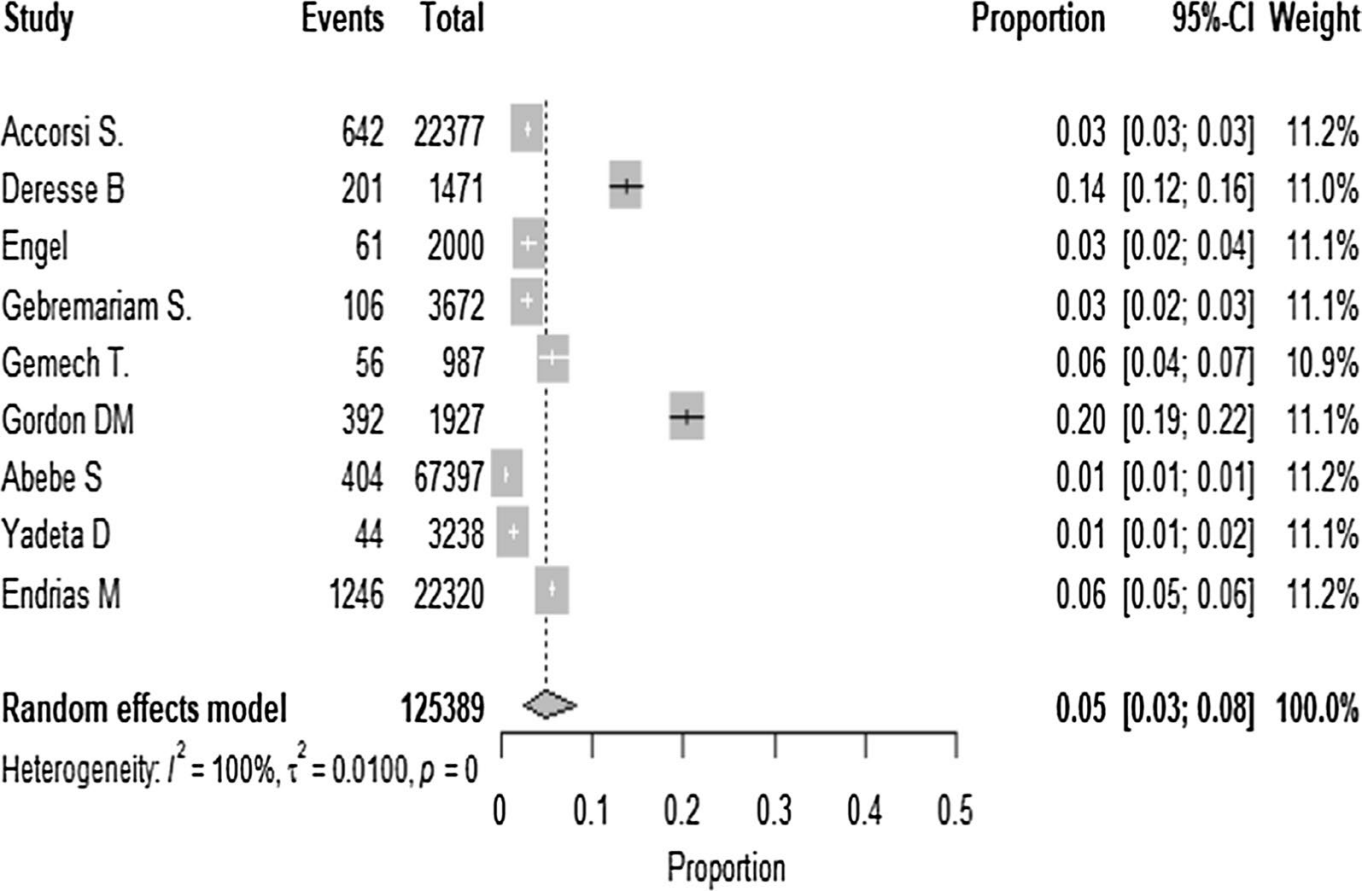
Forest plot for the prevalence of cardiovascular disease in Ethiopia

The estimated pooled prevalence of cardiovascular disease which was reported by nine studies using the fixed-effect model showed significant heterogeneity between the studies. As a result, the pooled prevalence was estimated using a random-effect model. Double arcsine transformation was used to normalize the distribution of the effect size. The review remarks that there is a high increment of CVD prevalence from a study done in 2008 to 2013, and it is declined in a 2015 study (Additional file 4). The prevalence of CVD ranges between 1 and 20%. %. In the random-effect model, the prevalence of CVD was 5% (95% CI: 3–8%) with significant heterogeneity between the studies (I^2^ = 99.75%), *p* < 0.001) (Fig. 2).

#### Subgroup analysis and investigation of heterogeneity

**Fig. 3:**
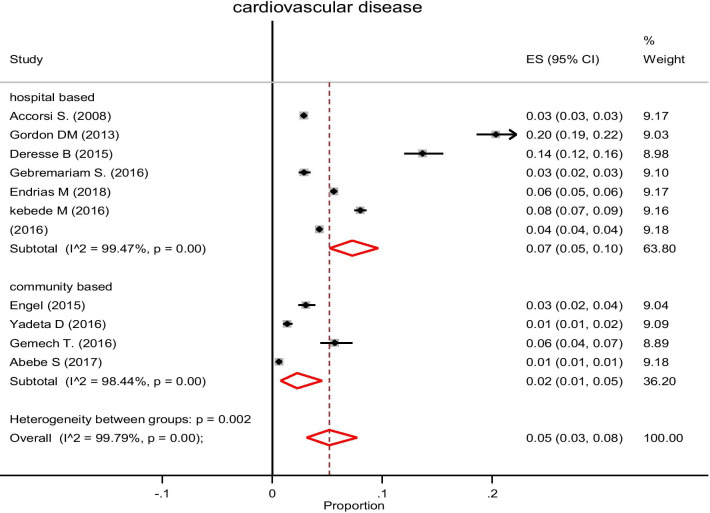
Subgroup analysis of cardiovascular disease prevalence by the source of population

Subgroup analysis by source population was conducted, and the prevalence of CVD was higher among the population who visited hospitals (8%, 95% CI: 4–12%) compared with the general population (2%, 95% CI: 1–5%). In the subpopulation analysis, potential heterogeneity was detected in the prevalence estimates of CVD across studies (I^2^ range: 98.4–99.6%; all *p* < 0.001) (Fig. 3).

We have also performed a subgroup meta-analysis based on the Ethiopian geographical region because of the overall prevalence difference across regions. Disease prevalence ranges between 3% [14, 15]and 6% [13] in Oromia, 1% [16] and 20% [17] in Amhara, and 6% [20] and 14% [21] SNNP regions (Additional file 5).

### Sex difference in the prevalence of CVD

Two hospital-based [15, 18] and two community-based [16, 19] studies with a total of 9,6684 (45,801 male and 50,883 female participants were included. In the hospital-based studies, the prevalence ranges from 1.8–3.9% to 1.9–2.2% for males and females, respectively. The general population (community-based) prevalence ranges from 0.3–1.1% to 0.9–1.7% for males and females, respectively.

The pooled prevalence of CVD among males and females were 2% (95% CI: 0–4%) and 2% (95% CI 1–3%; I^2^: 97.75–99.1, *p* = 0.00), respectively (Additional file 6).

### Sensitivity analysis

For further investigation about the source of potential heterogeneity in the prevalence, a sensitivity analysis was performed. After serially repeated exclusion of each study in the meta-analysis, the review revealed that two studies [19, 23] have been found to have an effect on the overall prevalence. These studies had an effect to vary by 1% above [21] and 1% below [16] for the overall prevalence of CVD.

### Association of sex and cardiovascular disease prevalence

**Fig. 4:**
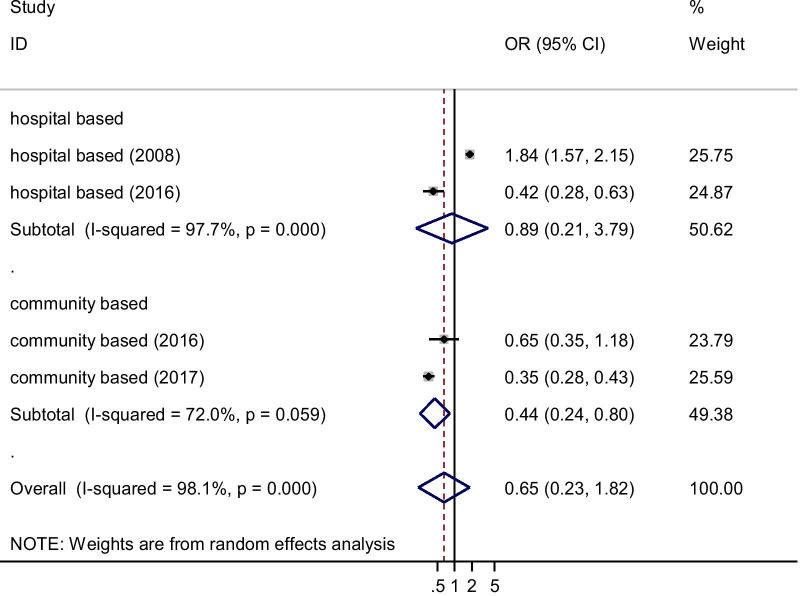
Forest plot of the risk of being male for cardiovascular disease: a meta-analysis

In this meta-analysis, only four studies were included. The pooled effect of being male was decreased by 35% (0.65, 95% CI: 0.23–1.82; I2 = 98.1%, *p* < 0.001) to develop CVD as compared with their counterparts (Fig. 4).

## Discussion

The prevalence of cardiovascular disease differed across studies. The current systematic review and meta-analysis incorporate nine studies to estimate the pooled prevalence of the cardiovascular disease. The overall prevalence of CVD was 5% (95% CI: 3, 8%). We have also quantified the prevalence based on the source of the population (hospital-based and community based). Significant heterogeneity was detected across studies for all these prevalence estimates, and the results were interpreted with caution.

In the current finding, we observed that there is a high increment of CVD prevalence from a study in 2008 to 2013, and it declined in a 2015 study, otherwise, no significant evidence of increment and/or decrement was observed in the prevalence of CVD across studies through time. However, other research outputs revealed that the trend of CVD and mortality attributed to CVD is increasing in Ethiopia [22, 23]. Additionally, a systematic research output which was conducted among sub-Saharan countries revealed that the prevalence of CVD and mortality due to CVD was not declined [24].

In the sub-group meta-analysis, the overall prevalence of CVD among individuals who visited or were admitted in hospitals was around four times higher as compared with the general population. The heterogeneity between studies for both groups was highly taking into consideration [25]. However, the pooled prevalence in the general population was lower than the prevalence in Gabon’s general population [26]. The population difference could contribute to the observed discrepancy; the participants included in the current study were all age groups whereas, in Gabon's study they were above 40 years of age. This indicates that elderly populations are susceptible to diabetes, hypertension, and obesity which are the risk factor of CVD [27, 28].

Based on the hospital and general population, the subgroup analysis showed that heterogeneity was highly concerned. It has been observed that the source of data being secondary data for hospital-based studies and primary data by interviewer-administered questionnaire for the general population (community-based studies) and variation of the age group for the included studies, were the possible source of heterogeneity.

Based on the geographical distribution, the highest prevalence in CVD was seen in SNNPs followed by the Oromia region. However, it was hard to say that the highest pooled prevalence was occurred in SNNP because of the high heterogeneity. In the Amhara region, there is a variation of CVD prevalence. The possible reason for this variation could be the population difference. In other words, the study by Gordon et al. considered the pediatric population only (median age 2.2 years) whereas the study done by Abebe et al. examined both pediatric and adult populations.

In the current study, the overall sex distribution of the disease among males and females was similar. This estimate was consistent with a report done in Gabon [26]. However, different literature [29, 30] suggests that males are at higher risk of having heart disease, but recent findings suggest that heart disease prevalence is increasing in middle age women while it is declining in males within the same age range [31]. The other explanation is that women develop CVD after 7 to 10 years older than males [31]. This might be due to the consequence of menopause transition which is related to increased heart disease risk [32] and in this review, women had a higher mean age than men.

Nevertheless, the prevalence was less in males than females in Mexico, China, India, Russian Federation, Ghana, and South Africa [33]. On the other hand, the overall meta-analysis report of sex effect on CVD showed that females were at high risk as compared with males. The study found that males had 35% less risk as compared with their counterparts. Our finding was supported by a study done in Southeast and West Asia, Nigeria, and Ghana [34].

By considering the source population, males from the general population were less likely to develop CVD compared with males from hospitals. Similarly, the prevalence of CVD among people who visit hospitals was higher (8%; 95% CI: 4- 12%) than the general population (2%; 95% CI: 1–5%). The plausible reason for this difference could be, in Ethiopia, males are involved in field activities whereas female's involvement in such activities is less. As a result, males can easily feel the disease which may increase their health-seeking behavior. Therefore, although there are contradicting finding as to the magnitude of CVD among males and females, due attention shall be given to the male population. Further, a well-designed original study is recommended in this regard.

Even though Ethiopia has nine regions, many of the studies included in the current review were from Oromia, Amhara, and SNNP. Furthermore, the number of studies included in this systematic review and meta-analysis were few. Since the included regions account for 80% of the population [35], understanding the review with caution would make to generalize for Ethiopian population.

### Strength and limitations of this review and meta-analysis

Performing quality assessment and data extraction by two reviewers to avoid the reviewer's bias is the strength of this study. In addition, subgroup and sensitivity analyses were performed to determine the effect of heterogeneity. However, we have found that heterogeneity was highly considerable, and the broad pooling of all cardiovascular disease that lacks detailed description for sub-types of cardiovascular disease to indicate the clinical and public health importance due to the small number of included studies.

## Conclusion

The prevalence of cardiovascular disease was high. A higher prevalence of CVD was found among patients who visited health institutions than the general population and no observed significant sex difference in the prevalence. Further studies are recommended to identify the determinants and consequences of CVD in Ethiopia.

## Supplementary Information

**Additional file 1:** Searching strategies.

**Additional file 2:** Risk of bias assesment.

**Additional file 3:** Assessment of publication bias using Egger’s test.

**Additional file 4:** Trend of CVD prevalence per year.

**Additional file 5:** The pooled prevalence of cardiovascular disease in Ethiopia by region.

**Additional file 6:** forest plot of prevalence of cardiovascular disease among males and female.

## Data Availability

The datasets supporting the conclusions of this article are included in the article.

## Abbreviations

CI: Confidence interval
CVD: Cardiovascular disease
CD: None communicable disease
ES: Effect size
SNNP: Southern nation nationality and people
OR: Odds ratio
WHO: World Health Organization
