# Supplementary material for: The prevalence of cardiovascular disease in Ethiopia: a systematic review and meta-analysis of institutional and community-based studies
Source: BMC Cardiovasc Disord. 2021 Jan 18;21:37. doi: 10.1186/s12872-020-01828-z (PMC7814574; doi:10.1186/s12872-020-01828-z)
Supplement: Supplementary file 1 — Additional file 1: Searching strategies. [file 12872_2020_1828_MOESM1_ESM.docx]

Appendix 1A

Searching strategy

**Keywords included in the search strategy for Scopus databases; terms searched for in the title, abstract and key words of papers**

| MeSH heading | Entry terms | Searching combination | Searching date | Total records |
| --- | --- | --- | --- | --- |
| Cardiovascular disease | Heart disease, vascular disease, cardiovascular abnormality , cardiovascular infection | ( TITLE-ABS-KEY ( "cardiovascular disease"  OR  " vascular disease"  OR  " cardiovascular abnormality"  OR  "cardiovascular infection"  OR  "heart disease" )  AND  ALL ( prevalence  OR  epidemiology  OR  "disease frequency" )  AND  TITLE-ABS KEY ( ethiopia ) )  AND  DOCTYPE ( ar )  AND  PUBYEAR  >  1999 | 6/16/2018 | 92 |
| Rheumatic heart disease | Bouillaud Disease,Bouillaud's Disease | ( TITLE-ABS-KEY ( "rheumatic heart disease"  OR  "Bouillaud Disease"  OR  "Bouillaud's Disease" )  AND  ALL  ( prevalence  OR  epidemiology  OR  "disease frequency" )  AND  TITLE-ABS KEY ( ethiopia ) )  AND  DOCTYPE ( ar )  AND  PUBYEAR  >  1999 | 6/16/2018 | 13 |
| Heart failure | Cardiac Failure, Congestive Heart Failure,  Heart Decomposition, Left-Sided heart failure,Right-Sided heart failure and  Myocardial Failure | ( TITLE-ABS-KEY ( "heart failure"  OR  "Cardiac Failure"  OR  "Congestive Heart Failure"  OR  "Heart Decompensation"  OR  "myocardial failure"  OR  "congestive heart failure" )  AND  ALL ( prevalence  OR  epidemiology  OR  "disease frequency" )  AND  TITLE-ABS-KEY ( ethiopia ) )  AND  DOCTYPE ( ar )  AND  PUBYEAR  >  1999 | 6/16/2018 | 19 |
| stroke | Apoplexy, CVA (Cerebrovascular Accident),  Cerebral Stroke ,Cerebrovascular Accident Cerebrovascular Apoplexy ,Cerebrovascular Stroke and Acute Vascular Accident | ( TITLE-ABS-KEY ( stroke  OR  apoplexy  OR  " cerebrovascular accident"  OR  "cerebral stroke"   OR  "cerebrovascular stroke"  OR  "cerebrovascular apoplexy"  OR  "vascular accident"  OR  " brain accident" )  AND  ALL ( prevalence  OR  epidemiology  OR  "disease frequency" )  AND  TITLE-ABS-KEY ( ethiopia ) )  AND  DOCTYPE ( ar )  AND  PUBYEAR  >  1999 | 6/16/2018 | 24 |
| Myocardial ischemia | Heart Disease, Ischemic,Ischemia, Myocardial and Ischemic Heart Disease | ( TITLE-ABS-KEY ( "myocardial ischemia"  OR  " heart disease"  OR  ischemia  OR  ischemic  OR  myocardial  OR  "ischemic heart disease" )  AND  ALL ( prevalence  OR  epidemiology  OR  "disease frequency" )  AND  TITLE-ABS-KEY ( ethiopia ) )  AND  DOCTYPE ( ar )  AND  PUBYEAR  >  1999 | 6/17/2018 | 46 |
| Hypertensive heart disease |  | ( TITLE-ABS-KEY ( "hypertensive heart disease" )  AND  ALL ( prevalence  OR  epidemiology  OR  "disease frequency" )  AND  TITLE-ABS-KEY ( ethiopia ) )  AND  DOCTYPE ( ar )  AND  PUBYEAR  >  1999 | 6/17/2018 | 3 |
| Coronary disease | Coronary heart disease | ( TITLE-ABS-KEY ( "coronary disease"  OR  "coronary heart disease"  OR  "coronary dis" )  AND  ALL ( prevalence  OR  epidemiology  OR  "disease frequency" )  AND  TITLE-ABS-KEY ( ethiopia ) )  AND  DOCTYPE ( ar )  AND  PUBYEAR  >  1999 | 6/17/2018 | 3 |

**Appendix 1B Keywords included in the search strategy for Midline/pub med databases; terms searched for in the title and abstract of papers**

| MeSH heading | Entry terms | Searching combination | Searching date | Total records |
| --- | --- | --- | --- | --- |
| Cardiovascular disease | Heart disease, vascular disease, cardiovascular abnormality , cardiovascular infection | ((("cardiovascular disease"[Title/Abstract] OR "vascular disease"[Title/Abstract] OR "heart disease"[Title/Abstract] OR " cardiovascular abnormality"[Title/Abstract] OR "cardiovascular infection"[Title/Abstract])) AND (prevalence OR epidemiology OR "disease frequency")) AND Ethiopia[Title/Abstract]  Filters: Publication date from 2000/01/01 to 2018/06/17 | 6/17/2018 | 44 |
| Rheumatic heart disease | Bouillaud Disease,Bouillaud's Disease | ((("rheumatic heart disease"[Title/Abstract] OR " Bouillaud Disease"[Title/Abstract] OR "Bouillaud's Disease"[Title/Abstract])) AND (prevalence OR epidemiology OR " disease frequency")) AND ethiopia[Title/Abstract]  Filters: Publication date from 2000/01/01 to 2018/06/17 | 6/17/2018 | 14 |
| Heart failure | Cardiac Failure, Congestive Heart Failure,  Heart Decomposition, Left-Sided heart failure,Right-Sided heart failure and  Myocardial Failure | (((" heart failure" [Title/Abstract] OR "congestive heart failure"[Title/Abstract] OR " heart decomposition"[Title/Abstract] OR " cardiac failure"[Title/Abstract] OR " myocardial failure"[Title/Abstract] OR " left sided heart failure"[Title/Abstract] OR "right sided heart failure"[Title/Abstract])) AND (prevalence OR epidemiology OR "disease frequency")) AND Ethiopia[Title/Abstract]  Filters: Publication date from 2000/01/01 to 2018/06/18 | 6/18/2018 | 16 |
| Stroke | Apoplexy, CVA (Cerebrovascular Accident),  Cerebral Stroke ,Cerebrovascular Accident Cerebrovascular Apoplexy ,Cerebrovascular Stroke and Acute Vascular Accident | (((stroke[Title/Abstract] OR apoplexy[Title/Abstract] OR "cerebrovascular accident"[Title/Abstract] OR "cerebrovascular stroke"[Title/Abstract] OR " cerebrovascular apoplexy"[Title/Abstract] OR "vascular accident"[Title/Abstract] OR "brain accident"[Title/Abstract])) AND (prevalence OR epidemiology OR "disease frequency")) AND Ethiopia[Title/Abstract]  Filters: Publication date from 2000/01/01 to 2018/06/18 |  | 13 |
| Myocardial ischemia | Heart Disease, Ischemic,Ischemia, Myocardial and Ischemic Heart Disease | ((("myocardial ischemia"[Title/Abstract] OR " heart disease"[Title/Abstract] OR ischemia[Title/Abstract] OR ischemic[Title/Abstract] OR myocardial[Title/Abstract] OR "ischemic heart disease"[Title/Abstract])) AND (prevalence OR epidemiology OR "disease frequency")) AND ethiopia[Title/Abstract] Filters: Publication date from 2000/01/01 to 2018/06/18 |  | 21 |
| Hypertensive heart disease |  | (("hypertensive heart disease"[Title/Abstract]) AND (prevalence OR epidemiology OR "disease frequency")) AND Ethiopia[Title/Abstract]  Filters: Publication date from 2000/01/01 to 2018/06/18 |  | 1 |
| Coronary disease | Coronary heart disease | ((("coronary disease"[Title/Abstract] OR " coronary heart disease"[Title/Abstract])) AND (prevalence OR epidemiology OR "disease frequency")) AND Ethiopia[Title/Abstract]  Filters: Publication date from 2000/01/01 to 2018/06/18 |  | 1 |
| **others** | | | | |
| google | | | 6/18/2018 | 10 |
| Google scholar | | | 6/18/2018 | 12 |
| Grey literature | | | 6/18/2018 | 2 |
