# Supplementary material for: The prevalence of cardiovascular disease in Ethiopia: a systematic review and meta-analysis of institutional and community-based studies
Source: BMC Cardiovasc Disord. 2021 Jan 18;21:37. doi: 10.1186/s12872-020-01828-z (PMC7814574; doi:10.1186/s12872-020-01828-z)
Supplement: Supplementary file 2 — Additional file 2: Risk of bias assesment. [file 12872_2020_1828_MOESM2_ESM.docx]

Additional file 2

Risk of bias assessment

The risk of bias of each study was evaluated using quality assessment checklist for prevalence studies

(Adapted from Hoy et al (14)). The overall risk of study bias has three categories 1) low risk (0-3), (2) moderate risk (4-6) finally high risk (7-9)

| **First Author (year)** | **ENTRY** | **JUDGEMENT** | **DESCRIPTION** |
| --- | --- | --- | --- |
| Accorsi (2008) | ***External Validity*** |  |  |
| **1** | Was the study’s target population **a close representation** of the national population in relation to relevant variables, e.g. age, sex, occupation? | **0** | **It adress all age groups and both sex populations visit in the hospital** |
| **2** | Was the sampling frame a **true or close representation** of the target population? | **0** | **The study samples were drawn from the target population** |
| **3** | Was some form of **random selection** used to select the sample, OR, was a census undertaken? | **0** | **They reviewed all discharge records** |
| **4** | Was the likelihood of **non-response bias** **minimal?** | **0** | **It doesn’t creat non response bias** |
|  | ***Internal Validity*** |  |  |
| **5** | Were data collected **directly from the subjects** (as opposed to a proxy)? | **1** | **Secondondary data( incomplete data may be matters)** |
| **6** | Was an acceptable case definition used in the study? | **0** | **There was clear case defination** |
| **7** | Was the study instrument that measured the parameter of interest (e.g. prevalence of low back pain) shown to have **reliability and validity (if necessary)**? | **1** | **Not well mentioned** |
| **8** | Was the **same mode of data collection** used for all subjects? | **0** | **Use similar mode of data collection techniques** |
| **9** | Were the **numerator(s) and denominator(s)** for the parameter of interest appropriate? | **0** | **Both numerator and denominator were used appropriatly** |
|  | Overall risk of Bias | **2- low risk** |  |
| **First Author (year)** | ENTRY | **JUDGEMENT** | **DESCRIPTION** |
| Deresse (2015) | External Validity |  |  |
| **1** | Was the study’s target population a close representation of the national population in relation to relevant variables, e.g. age, sex, occupation? | **1** | **All individuals age 15 and above included in the study but the relevant variable sex** |
| **2** | Was the sampling frame a true or close representation of the target population? | **0** | **The study samples were drawn from the target population** |
| **3** | Was some form of random selection used to select the sample, OR, was a census undertaken? | **0** | **Census was under taken** |
| **4** | Was the likelihood of non-response bias minimal? | **1** | **The response rate for the study was above 82%** |
|  | Internal Validity |  |  |
| **5** | Were data collected directly from the subjects (as opposed to a proxy)? | **0** | **the data was collected directly from admitted patients** |
| **6** | Was an acceptable case definition used in the study? | **0** | **There was clear case defination** |
| **7** | Was the study instrument that measured the parameter of interest (e.g. prevalence of low back pain) shown to have reliability and validity (if necessary)? | **0** | **The researcher used national institute of health stroke scale** |
| **8** | Was the same mode of data collection used for all subjects? | **0** | **Use similar mode of data collection techniques** |
| **9** | Were the numerator(s) and denominator(s) for the parameter of interest appropriate? | **0** | **Both numerator and denominator were used appropriatly** |
|  | Overall risk of Bias | **2 (low risk)** |  |

| **First Author (year)** | **ENTRY** | **JUDGEMENT** | **DESCRIPTION** |
| --- | --- | --- | --- |
| Engel (2015) | ***External Validity*** |  |  |
| **1** | Was the study’s target population **a close representation** of the national population in relation to relevant variables, e.g. age, sex, occupation? | **1** | The study was conducted in one area only and relevant variables were not incorporated |
| **2** | Was the sampling frame a **true or close representation** of the target population? | **1** | The sampling frame was a list of one particular area within the overall target population |
| **3** | Was some form of **random selection** used to select the sample, OR, was a census undertaken? | **0** | The study uses appropriate sampling methods |
| **4** | Was the likelihood of **non-response bias** **minimal?** | **0** | 99% response rate |
|  | ***Internal Validity*** |  |  |
| **5** | Were data collected **directly from the subjects** (as opposed to a proxy)? | **0** | data collected **directly from the subjects** |
| **6** | Was an acceptable case definition used in the study? | **0** | acceptable case definition were used in the document |
| **7** | Was the study instrument that measured the parameter of interest (e.g. prevalence of low back pain) shown to have **reliability and validity (if necessary)**? | **1** | The study was assessing only prevalence so need of validated instrument |
| **8** | Was the **same mode of data collection** used for all subjects? | **0** | **same mode of data collection** used for all subjects |
| **9** | Were the **numerator(s) and denominator(s)** for the parameter of interest appropriate? | **0** | Yes, appropriate parameters were used |
|  | Overall risk of Bias | **3- low risk** |  |
| **First Author (year)** | ENTRY | **JUDGEMENT** | **DESCRIPTION** |
| Gebremariam (2016) | External Validity |  |  |
| **1** | Was the study’s target population a close representation of the national population in relation to relevant variables, e.g. age, sex, occupation? | **0** | All eligible individuals with relevan variables |
| **2** | Was the sampling frame a true or close representation of the target population? | **1** | **unclear** |
| **3** | Was some form of random selection used to select the sample, OR, was a census undertaken? | **1** | They use non probable sampling which is consicative |
| **4** | Was the likelihood of non-response bias minimal? | **0** | 100% as it is consecutive |
|  | Internal Validity |  |  |
| **5** | Were data collected directly from the subjects (as opposed to a proxy)? | **1** | Data were extracted from the patient charts and were filled into the questionnaire format by the investigators |
| **6** | Was an acceptable case definition used in the study? | **0** | Heart failure ..Based on standard definition ….NYHA classes III and IV  Diagnosis of congenital as well as acquired heart disease is confrmed by echocardiographic examination (2D, M-mode, and color-doppler mode imaging). |
| **7** | Was the study instrument that measured the parameter of interest (e.g. prevalence of low back pain) shown to have reliability and validity (if necessary)? | **0** | Pretested |
| **8** | Was the same mode of data collection used for all subjects? | **1** | All data were from patient chart |
| **9** | Were the numerator(s) and denominator(s) for the parameter of interest appropriate? | **0** | Yes, appropriate parameters were used |
|  | Overall risk of Bias | **Moderate risk** | **More than 3 domians were at high risk** |

| **First Author (year)** | **ENTRY** | **JUDGEMENT** | **DESCRIPTION** |
| --- | --- | --- | --- |
| Gemech T (2016) | ***External Validity*** |  |  |
| **1** | Was the study’s target population **a close representation** of the national population in relation to relevant variables, e.g. age, sex, occupation? | **1** | Only 6-25 years of age included |
| **2** | Was the sampling frame a **true or close representation** of the target population? | **1** | Only one zone cannot represent rural Ethiopia |
| **3** | Was some form of **random selection** used to select the sample, OR, was a census undertaken? | **1** | Initially purposive then systematic and no census |
| **4** | Was the likelihood of **non-response bias** **minimal?** | **0** | 82% RR |
|  | ***Internal Validity*** |  |  |
| **5** | Were data collected **directly from the subjects** (as opposed to a proxy)? | **0** | Yes |
| **6** | Was an acceptable case definition used in the study? | **0** | RHD on the basis of the 2012 WHF criteria |
| **7** | Was the study instrument that measured the parameter of interest (e.g. prevalence of low back pain) shown to have **reliability and validity (if necessary)**? | **1** | Tool not pretested |
| **8** | Was the **same mode of data collection** used for all subjects? | **0** | All eligible subjects had a face-to-face interview |
| **9** | Were the **numerator(s) and denominator(s)** for the parameter of interest appropriate? | **0** |  |
|  | Overall risk of Bias | **4- moderate risk** |  |
| **First Author (year)** | ENTRY | **JUDGEMENT** | **DESCRIPTION** |
| Gordon DM (2013) | External Validity |  |  |
| **1** | Was the study’s target population a close representation of the national population in relation to relevant variables, e.g. age, sex, occupation? | **0** | **The researcher accounts all pediatric ages, both sex** |
| **2** | Was the sampling frame a true or close representation of the target population? | **0** | **It accounts all pediatric individuals recorded in the hospital** |
| **3** | Was some form of random selection used to select the sample, OR, was a census undertaken? | **0** | **It was censu** |
| **4** | Was the likelihood of non-response bias minimal? | **0** | **It was secondary data and complete census** |
|  | Internal Validity |  |  |
| **5** | Were data collected directly from the subjects (as opposed to a proxy)? | **1** | **Simply reviewing secondary data** |
| **6** | Was an acceptable case definition used in the study? | **0** | **There was clear case defination** |
| **7** | Was the study instrument that measured the parameter of interest (e.g. prevalence of low back pain) shown to have reliability and validity (if necessary)? | **1** | **There is no clear report** |
| **8** | Was the same mode of data collection used for all subjects? | **0** | **Note applicable** |
| **9** | Were the numerator(s) and denominator(s) for the parameter of interest appropriate? | **0** | Appropriate numerator and denominator was used to calculate prevalence |
|  | Overall risk of Bias | **2-low risk** |  |

| **First Author (year)** | **ENTRY** | **JUDGEMENT** | **DESCRIPTION** |
| --- | --- | --- | --- |
| Abebe S (2017) | ***External Validity*** |  |  |
| **1** | Was the study’s target population **a close representation** of the national population in relation to relevant variables, e.g. age, sex, occupation? | **0** | **It accunts all relevant variables in both rural and urban** |
| **2** | Was the sampling frame a **true or close representation** of the target population? | **0** | **The sampling from was a list of all populations in that target population** |
| **3** | Was some form of **random selection** used to select the sample, OR, was a census undertaken? | **0** | **It was simple random sampling technique** |
| **4** | Was the likelihood of **non-response bias** **minimal?** | **1** | **Not stated** |
|  | ***Internal Validity*** |  |  |
| **5** | Were data collected **directly from the subjects** (as opposed to a proxy)? | **0** | **Data was collected directly from study subjects through self addminister questions not from others** |
| **6** | Was an acceptable case definition used in the study? | **0** | **There was clear case defination** |
| **7** | Was the study instrument that measured the parameter of interest (e.g. prevalence of low back pain) shown to have **reliability and validity (if necessary)**? | **0** | **The questionare were tested to assess the prevalence of CVD in the dabat research center** |
| **8** | Was the **same mode of data collection** used for all subjects? | **0** | **Information was collected through self administer questions** |
| **9** | Were the **numerator(s) and denominator(s)** for the parameter of interest appropriate? | **0** | Appropriate numerator and denominator were used to calculate prevalence |
|  | Overall risk of Bias | **1 (low risk)** |  |
| **First Author (year)** | ENTRY | **JUDGEMENT** | **DESCRIPTION** |
| Yadeta (2016) | External Validity |  |  |
| **1** | Was the study’s target population a close representation of the national population in relation to relevant variables, e.g. age, sex, occupation? | **0** | Includes all regions of Ethiopia with relevant variables |
| **2** | Was the sampling frame a true or close representation of the target population? | **0** | a list of almost every individual within the target population are included in the sampling frame |
| **3** | Was some form of random selection used to select the sample, OR, was a census undertaken? | **0** | The study try to sample every individual with in the target population |
| **4** | Was the likelihood of non-response bias minimal? | **0** | The response rate was 98.1% |
|  | Internal Validity |  |  |
| **5** | Were data collected directly from the subjects (as opposed to a proxy)? | **0** | Data were collected directly from the study subjects |
| **6** | Was an acceptable case definition used in the study? | **0** | Used standard case definitions |
| **7** | Was the study instrument that measured the parameter of interest (e.g. prevalence of low back pain) shown to have reliability and validity (if necessary)? | **1** | Instrument was not validated or reliability test were not done |
| **8** | Was the same mode of data collection used for all subjects? | **0** | **same mode of data collection** used for all subjects |
| **9** | Were the numerator(s) and denominator(s) for the parameter of interest appropriate? | **0** | Appropriate numerator and denominator was used to calculate prevalence |
|  | Overall risk of Bias | **1 (low risk)** |  |

| **First Author (year)** | **ENTRY** | **JUDGEMENT** | **DESCRIPTION** |
| --- | --- | --- | --- |
| Endrias (2018) | ***External Validity*** |  |  |
| **1** | Was the study’s target population **a close representation** of the national population in relation to relevant variables, e.g. age, sex, occupation? | **0** | **Incorporate all age and both sex** |
| **2** | Was the sampling frame a **true or close representation** of the target population? | **0** | a list of almost every individual within the target population are included in the sampling frame then the researcher retrieved from the record |
| **3** | Was some form of **random selection** used to select the sample, OR, was a census undertaken? | **0** | The study try to sample every individual with in the target population |
| **4** | Was the likelihood of **non-response bias** **minimal?** | **0** | **Unlikely because it was retrived using secondary data** |
|  | ***Internal Validity*** |  |  |
| **5** | Were data collected **directly from the subjects** (as opposed to a proxy)? | **0** | **Not applicable (the data were retrived from secondary data)** |
| **6** | Was an acceptable case definition used in the study? | **1** | **There is no clear statement of case defination on CVD** |
| **7** | Was the study instrument that measured the parameter of interest (e.g. prevalence of low back pain) shown to have **reliability and validity (if necessary)**? | **1** | **There was no clear report on reliability and validity of the nstrument** |
| **8** | Was the **same mode of data collection** used for all subjects? | **0** | **Retrived from secondary data** |
| **9** | Were the **numerator(s) and denominator(s)** for the parameter of interest appropriate? | **0** | **Appropriate numrator and denominator were used** |
|  | Overall risk of Bias | **1 (low risk)** |  |
