# Supplementary material for: The prevalence of cardiovascular disease in Ethiopia: a systematic review and meta-analysis of institutional and community-based studies
Source: BMC Cardiovasc Disord. 2021 Jan 18;21:37. doi: 10.1186/s12872-020-01828-z (PMC7814574; doi:10.1186/s12872-020-01828-z)
Supplement: Supplementary file 3 — Additional file 3: Assessment of publication bias using Egger’s test. [file 12872_2020_1828_MOESM3_ESM.docx]

Additional file 3 - assessment of publication bias using egger’s test

Std_Eff Coef. Std. Err. t P>t [95% Conf. Interval]

slope 84.09943 66.64458 1.26 0.247 -73.48995 241.6888

bias 0.0305054 .0328128 0.93 0.383 .0270846 .1080953
