# Supplementary material for: The prevalence of cardiovascular disease in Ethiopia: a systematic review and meta-analysis of institutional and community-based studies
Source: BMC Cardiovasc Disord. 2021 Jan 18;21:37. doi: 10.1186/s12872-020-01828-z (PMC7814574; doi:10.1186/s12872-020-01828-z)
Supplement: Supplementary file 4 — Additional file 4: Trend of CVD prevalence per year. [file 12872_2020_1828_MOESM4_ESM.docx]

Figure. Trends of CVD prevalence through years of publication.
