# Supplementary material for: The prevalence of cardiovascular disease in Ethiopia: a systematic review and meta-analysis of institutional and community-based studies
Source: BMC Cardiovasc Disord. 2021 Jan 18;21:37. doi: 10.1186/s12872-020-01828-z (PMC7814574; doi:10.1186/s12872-020-01828-z)
Supplement: Supplementary file 6 — Additional file 6: forest plot of prevalence of cardiovascular disease among males and female. [file 12872_2020_1828_MOESM6_ESM.docx]

Additional file 5

Fig . forest plot of prevalence of cardiovascular disease among males

Fig . forest plot of prevalence of cardiovascular disease among females
